# Impact of the production method on the properties of erythrocyte-derived extracellular particles: a quantitative and qualitative evaluation

**DOI:** 10.7150/ntno.116627

**Published:** 2025-10-24

**Authors:** Maria Chiara Ciferri, Nicole Grinovero, Maria Cristina Gagliani, Katia Cortese, Chiara Romiti, Alessia Brossa, Cristina Grange, Benedetta Bussolati, Gianluca Ubezio, Vanessa Agostini, Andrea Petretto, Rodolfo Quarto, Ulla Impola, Saara Laitinen, Roberta Tasso

**Affiliations:** 1 Department of Experimental Medicine (DIMES), University of Genova, Genova, Italy.; 2 Core Facility for Omics Science, IRCCS Istituto G. Gaslini, Genova, Italy.; 3 Department of Molecular Biotechnology and Health Sciences, University of Turin, Turin, Italy.; 4 Department of Medical Biotechnologies, University of Siena, Siena, Italy.; 5 Department of Medical Sciences, University of Turin, Turin, Italy.; 6 IRCCS Ospedale Policlinico San Martino, Genova, Italy.; 7 Research and Development, Finnish Red Cross Blood Service, Vantaa, Finland.

**Keywords:** Natural nanoparticles, Erythrocytes-derived extracellular particles, extracellular particles isolation, targeted drug delivery, proteomic signature

## Abstract

Red blood cell-derived extracellular particles (RBCEPs) possess many benefits in healthcare applications representing a simple and powerful platform for drug delivery. Nowadays, whether the different methods proposed to produce them could influence their properties remains poorly investigated. We compared three main types of RBCEPs: (i) naturally released during the blood bag storage (EryErythrosomes, EryEs), (ii) produced artificially through RBC sonication (NanoErythrosomes, NanoEs), and (iii) released after RBC chemical stimulation (Vesiculation-Induced EryEs, VI-EryEs). Concentration, protein amount, size distribution, and morphology (TEM) were preliminary evaluated. Then the expression of EV- and erythrocyte-associated markers was assessed by flow cytometry and western blot while proteomic analyses estimated the differences in total protein content. The efficiency of membrane functionalization was assessed by copper-free click chemistry. All three subpopulations were able to be produced on a large scale, with NanoEs being the ones with the highest concentration, polydispersity, and largest dimension. The traditional EV-associated rounded shape was found to coexist with a tubular shape. Tetraspanin expression was absent while all three formulations expressed Alix, Flotillin-1, and Tsg101. On the other hand, Glycophorin A was the only RBC marker expressed ubiquitously. The analyzed subpopulations showed three distinct proteomic signatures which defined distinct enrichment profiles of molecular functions. Finally, NanoEs turned out to be the most efficient in surface functionalization adopting a copper-free click chemistry strategy. This work provides a pioneering contribution to the study and application of RBCEPs, indicating that the isolation method can significantly impact both the quantitative and qualitative properties of these natural nanoparticles.

## Introduction

Red blood cells (RBCs) are a valuable source for particle production due to their unique advantages. Comprising 70% of human cells, they are easily obtained, and thanks to the absence of nuclei and mitochondria the risk of gene transfer is reduced 1,2. RBC-derived extracellular particle (RBCEP) formulations offer significant benefits, such as scalability, cost-effectiveness, and reduced contamination risk 3. RBCEPs are naturally released in RBC concentrates 4 and play key roles in RBC turnover 5-7, immune modulation 8, and homeostasis 9. They also serve as efficient drug delivery systems, with the potential to be used in allogeneic settings, especially when derived from the O blood group 6, 10. Their ability to deliver therapeutic molecules such as miRNA 11-13 and to be conjugated with targeting molecules 3,14,15 has already been demonstrated.

Several effective methods have been proposed over the years to isolate particles from RBCs, giving rise to different RBCEP formulations. A primary distinction to be made involves categorizing particles (i) naturally produced by RBCs during their storage 16, and (ii) released upon RBC stimulation. Multiple vesiculation-inducing stimuli have been proven to be efficient for RBCEP production. They can be mainly divided into chemical and physical stimuli 6. Chemicals induce vesiculation by calcium channels and protein kinase C activation, leading to phosphatidylserine (PS) exposure and particle formation 6. Among the physical strategies, extrusion is one of the most commonly used techniques, alongside shear stress-based methods 9. From a functional perspective, the efficiency of each isolation protocol relies on its ability to scale up the RBCEP yield. Indeed, whether and how the different types of stimuli can affect RBCEP properties and composition remains unclear and poorly investigated. While RBCEP formulations are primarily used in drug delivery, there is no consensus on the optimal formulation for this purpose. This study compares three types of RBC-derived particles: naturally released EPs (EryErythrosomes, EryEs), sonication-induced EV-mimetics (NanoErythrosomes, NanoEs), and chemically induced EPs (vesiculation-induced EryErythrosomes, VI-EryEs). All three groups will be categorized as RBCEPs throughout the text.

This study has two main objectives: (i) to assess whether different isolation strategies affect the quantitative and qualitative characteristics of RBCEP formulations, and (ii) to evaluate which formulation is best suited for surface functionalization and efficient cellular uptake, key prerequisites for potential drug delivery applications.

Particles were characterized by concentration, size distribution, zeta potential, morphology, EV and RBC markers, and proteomic profiles. Membrane functionalization was performed using a copper-free click chemistry strategy to evaluate their potential as targeted drug delivery carriers. Finally, the internalization rates of each particle type were assessed in fibroblasts and THP-1 macrophages. Our findings suggest that NanoEs exhibit the most favorable properties for being applied as nanocarriers in targeted therapeutic applications.

## Methods

### RBC bag collection

Donor-derived standard leukocyte-reduced erythrocyte concentrates were provided from the Institutional Transfusion Center of Ospedale Policlinico San Martino, Genova. All concentrates were handled anonymously and only concentrates that could not be administered clinically were used just before the expiration date. The time that occurs from the blood bag preparation to the expiry is 42 days. Blood components were prepared according to the Council of Europe Guide to the Preparation, Use and Quality Assurance of Blood Components (EDQM 22nd Edition). Red blood cell units eliminated due to expiration were evaluated starting from the day after their validity expired.

All blood donors signed an informed consent, which includes the possibility of allocating part of the donation for research purposes, in accordance with the provisions of the ministerial decree.

### RBCEP isolation methods

All three RBCEP formulations were isolated from RBC units stored for 42 days. NanoEs and EryEs were isolated using previously developed protocols 17 with minor modifications. For the isolation of VI-EryEs, we employed a published method 3, incorporating a few adjustments. The initial “cleaning” phase is common to all the protocols 17. Each RBC concentrate (250 mL) was diluted 1:1 with calcium- and magnesium-free 1X phosphate-buffered saline (PBS) and centrifuged at 800×g for 10 min at RT. The supernatant containing EryEs was collected, while the pellet was resuspended in cold PBS and centrifuged at 1560×g for 20 min at RT. The supernatant was combined with the previous one, and the centrifugation step was repeated.

For EryEs isolation, the collected supernatants from the two initial centrifugations were subjected to ultracentrifugation (Optima XPN-100, Beckman Coulter) at 100 000×g for 60 minutes at 4°C with an SW28 swinging rotor. The pellets were then pooled and centrifuged for 1.5 h at 100 000×g (4°C) with a SW41Ti swinging rotor. After this last washing step, the pellets were suspended in PBS (final volume depending on the pellet amount), vortexed for 30 s, and centrifuged at 3000×g for 10' to remove any left contaminant.

For NanoEs isolation, the supernatants were discarded, and 15 mL of erythrocytes were sonicated at maximum power for 30s using a probe sonicator (UP 50H, Dr. Hielscher). The suspension was diluted with cold PBS and centrifuged at 1560×g for 20 min to remove cells and larger fragments. The supernatant was transferred into 6 ultracentrifuge tubes (0.5 mL/tube), filled with PBS, and ultracentrifuged at 100,000×g for 60 min at 4°C. Supernatants were discarded, pellets resuspended and centrifuged again at 100,000×g for 60 min at 4°C. The final pellets were suspended in PBS, vortexed, and centrifuged at 3000×g for 10 min to remove contaminants.

For VI-EryEs isolation, 20 mL of RBCs were diluted 3x with 0.1 mg/mL calcium chloride in PBS and incubated overnight at 37°C with 10 µM calcium ionophore. RBCs and cell debris were removed by serial centrifugation at 600×g for 20 min, 1600×g for 15 min, 3200×g for 15 min (Eppendorf Centrifuge 5810R, A-4-62 rotor) and 10,000×g for 30 min (SW28 swinging rotor) at 4°C. After filtration through a 0.45 µm filter, the supernatant was ultracentrifuged at 50,000×g for 1 h at 4°C. The pellet was resuspended in PBS and layered onto a 60% sucrose cushion, then ultracentrifuged at 50,000×g for 16 h at 4°C. The red layer above the sucrose was collected, washed at 50,000×g for 1 h at 4°C, and suspended in PBS.

All the quantitative and qualitative analyses were performed on fresh samples for each particle population. After all the qualitative and qualitative analyses were completed, the samples were aliquoted and frozen by immersing the vials for 30 s in liquid nitrogen before preserving them at -80°C for other uses. Uptake analysis was performed with thawed particles.

### Nanoparticles tracking analysis (NTA)

NTA analysis was conducted using the NanoSight NS300 instrument (Malvern Panalytical, UK), equipped with a 488nm laser and a high-sensitivity CCD camera. All EV samples were diluted in filtered PBS-EDTA to a final volume of 1 mL. Ideal measurement concentrations were found by pre-testing the ideal particle per frame value (20-100 particles/frame). The following settings were set according to the manufacturer's software manual (NanoSight NS300 User Manual). The camera level was increased until all particles were distinctly visible, not exceeding a particle signal saturation of over 20%. The ideal detection threshold was determined to include as many particles as possible with the restrictions that 10-100 red crosses were counted while only <10% were not associated with distinct particles. Blue cross count was limited to 5. Autofocus was adjusted so that indistinct particles were avoided. For each measurement, five 1-minute videos were captured under the following conditions: cell temperature: 25°C; syringe speed: 40 µl/s. After capture, the videos have been analyzed by NanoSight Software NTA 3.1 Build 3.1.46 with a detection threshold of 5. Hardware: embedded laser: 45 mW at 488 nm; camera: sCMOS. The number of completed tracks in NTA measurements was always greater than the proposed minimum of 1000 in order to minimize data skewing based on single large particles. The span, calculated as (D90 - D10) / D50, has been used for quantifying polydispersity and width of the size distribution.

### Bicinchoninic acid protein assay (BCA)

Pierce™ BCA Protein Assay Kit was used to evaluate protein concentration in each RBCEP subpopulation. Standards and working solutions were prepared following manufacturer instructions. An enhanced protocol with a microplate procedure was applied and the absorbance at 562 nm was measured with a spectrophotometer (SpectroStar).

### Zeta potential measurements

The zeta potential of the RBCEPs was measured Malvern Zetasizer Nano ZS (Malvern Panalytical, Malvern, UK) instrument. Briefly, the nanoparticles were diluted (1:65) in NaCl 10 mM solution and measured (3 repetitions, 30 measures) in duplicate.

### Transmission electron microscopy (TEM)

Twenty microliters of each RBCEP suspension were fixed by adding an equal volume of 2% paraformaldehyde in 0.1 mol/l phosphate buffer (pH 7.4), as previously described 18. EVs were then absorbed for 10 min to formvar-carbon coated copper grids by floating the grids on 5 μl drops on parafilm. Subsequently, grids with adhered vesicles were rinsed in PBS and negatively stained with 2% uranyl acetate for 5 min at room temperature. Stained grids were embedded in 2.5% methylcellulose for improved preservation and air-dried before examination. Electron micrographs were taken at Hitachi TEM microscope (HT7800 series, Tokyo, Japan) equipped with Megaview 3 digital camera and Radius software (EMSIS, Germany). To visualize EV size distribution, the results were plotted as a colorblind safe scatter dot plot in which each size measured is represented as a point along with lines for the median value and the range.

### Non-conventional flow cytometry

RBCEPs were characterized by non-conventional flow cytometry, as previously described 19. Briefly, 1 ×10⁹ particles in a final volume of 100 µL of PBS were stained with 1 µM CFDA-SE (CFSE) (Vybrant™ CFDA-SE Cell Tracer Kit, Thermo Fisher Scientific, Waltham, MA, USA) at either 4°C as a control to verify CFSE specificity and set the correct dimensional gate or 37°C for 20 min to visualize only intact particles. The expression of CD9 (APC Mouse Anti-Human CD9, 312108, BioLegend), CD63 (PE-Cy7 Mouse Anti-Human CD63, 561982, BD Biosciences), CD81 (BV421 Mouse Anti-Human CD81, 740079, BD Biosciences) and CD235a (APC Mouse Anti-Human CD235a, 551336, BD Biosciences) was evaluated within the CFSE positive events and compared to the corresponding isotype controls. Samples were acquired using a CytoFLEX S flow cytometer (Beckman Coulter) and collected data were analyzed using the FlowJo_v 10.9.0 software.

### Western blot

Each of the samples analyzed by WB was prepared as follows: 50 µLof particle suspension after isolation was mixed with 10X RIPA buffer (Cell Signaling) and 100X inhibitor proteases cocktail (Cell Signaling). Protein content was then quantified by Bicinchoninic acid (BCA) assay (Thermo Fisher Scientific). Ten μg of proteins for each sample were loaded on 4%-12% NuPAGE Bis-Tris gel (Thermo Fisher Scientific) after being mixed with NuPAGE Sample Reducing Agent 10X (Invitrogen) and NuPage LDS Sample Buffer 4X (Invitrogen) and heated at 60°C for 10 min. Electrophoresis was performed at 160 V and proteins were blotted on a polyvinylidene fluoride membrane for 1 h at 30V with an XCell Blot Module (Invitrogen). After blocking nonspecific sites with 5% non-fat dry milk (EuroClone, Italy) in Tris Buffered Saline with Tween 20 (TTBS, 20 mM Tris pH 7.5, 500 mM NaCl, 0.05% Tween 20), the membrane was incubated overnight at 4 °C with specific primary antibodies for: Flotillin-1 1:10 000 (ab133497, Abcam), Alix 1:1000 (ab186429, Abcam), TSG-101 1:1000 (T5701, Sigma), Glycophorin A 1:1000 (ab129024, Abcam), BAND 3/AE 1 1:1000 (ab77236, Abcam) diluted in 2.5% non-fat dry milk/TTBS. After washing three times with TTBS, membranes were incubated with specific HRP-conjugated secondary antibodies anti-mouse (Anti-mouse IgG HRP-linked Antibody, 7076S, Cell Signaling) or anti-rabbit (Anti-rabbit IgG HRP-linked Antibody, 7074S, Cell Signaling). Positivity was highlighted by providing the substrates for the chemiluminescence reaction of HRP (LiteAblot TURBO, EMP012001, Euroclone) and analyzed by UVITEC technology (chemiluminescence).

### RBCEP surface functionalization by copper-free click chemistry

Particles (1×10¹¹) were resuspended in 100 µL of PBS and stained with 1 µM CFSE and incubated at 37°C for 20 min. Then, following a previously described protocol 20 samples were mixed for 1 h at RT with 1 μg of DBCO-NHS ester (Dibenzocyclooctyne-N-hydroxy succinimidyl ester, 761524, Sigma). After the first incubation, 2 μl of a 5.9 mM (final concentration 56 μM) AlexaFluor647-azide (AZ-647) solution (A10277, ThermoFisher) was added to the reaction and incubated for 4 h at RT to obtain AF647-Click-particles. The reaction mixture without DBCO-NHS ester was considered a negative control. After the last incubation, samples were washed by SEC to remove unlabeled chemicals and analyzed by non-conventional flow cytometry considering AF647-Click particles those event APC positive within the CFSE positive events.

### Total reactive oxygen species (ROS) assay

MRC-5 (a fibroblast cell line) were selected as recipient cells for evaluating ROS production after RBCEP treatment. The following conditions were evaluated: untreated cells (basal ROS production, negative control), cells treated with 1 mM H_2_O_2_ (positive control), and cells treated with RBCEP formulations. MRC-5 were seeded in a 48-multiwell plate (12500 cells/well) and cultured in DMEM medium supplemented with 10% FBS, 1% pen-strep and 1% L-Glutamine. After 24 hours, NanoEs, EryEs and VI-EryEs were added in a serum-free media at two different concentrations (5*10^8^/mL and 5*10^9^/mL) for 16 h. A Total Reactive Oxygen Species Assay Kit 520nm (Invitrogen) was used for identifying the produced ROS. According to manufacturer instruction, after incubation with nanoparticles, ROS Assay Stain Solution 1X was added to cells for 1 h in serum-free media. A blank control, which was not stained with ROS Stain Solution was also included. Next, after removing the solution and washing, cells were treated with 1 mM H_2_O_2_ in serum-free for 2 h. The parallel wells containing the other conditions were instead incubated with serum-free media. Finally, cells were detached and analyzed by flow cytometry.

### RBCEP internalization by responder cells

THP-1 and MRC-5 cell lines were selected as responder cells to evaluate the internalization of the three RBCEP subpopulations. THP-1 were seeded in a 24-multiwell plate (400,000 cells/well) and cultured for 48 h in RPMI medium supplemented with 10% FBS, 1% pen-strep, 1% L-Glutamine and 100 ng/mL of phorbol-12-myristate-13-acetate (PMA) 21,22. For MRC-5, 60,000 cells/well were seeded in a 24-multiwell plate 48h before the experiment and cultured in DMEM medium supplemented with 10% of FBS, 1% pen-strep and 1% L-Glutamine. All three RBCEP populations were stained with 20 µM CellTrace™ Far Red Cell Proliferation Kit (ThermoFisher) for 1 h at 37°C. After that samples were washed by SEC and analyzed by both NTA (particle concentration) and flow cytometry (percentage of APC positive particles) before adding to cells.

### Mass spectrometry-based proteomics

#### Sample preparation

EV pellets were lysed, and proteins were denatured, reduced, and alkylated with 50 µL of iST-LYSE buffer (PreOmics) for 10 minutes at 95°C on an Eppendorf ThermoMixer (1000 rpm). Protein Aggregation Capture (PAC)-based protein isolation and digestion 23 were automated on a KingFisher™ Apex magnetic handling station (Thermo Scientific) in a 96-well format. Briefly, a 1:1 mixture of Sera-Mag™ SpeedBead Carboxylate Modified Magnetic Particles (Cytiva, 45152105050250 and 65152105050250) was added to lysed samples in plate #1, maintaining a protein-to-bead ratio of 1:4 (w/w). Proteins were precipitated onto beads by adding acetonitrile (ACN) to a final concentration of 70%, followed by two cycles of 1-minute mixing at medium speed and 10-minute pauses. Aggregated proteins on beads underwent sequential washes to remove contaminants, with three washes in 100% ACN, followed by 70% ethanol and isopropanol (plates #2-6). Each wash step was performed for 2.5 minutes at slow speed, with beads retained on the magnetic rack. On-bead digestion was carried out in 100 µL of 25 mM Tris buffer (pH 8.0), containing 0.7 µg of trypsin (Promega) and 0.3 µg of Lys-C (Wako) in plate #7 at 37°C for 2.5 hours. Post-digestion, the magnetic beads were separated from the digested samples and placed back into plate #1. Protease activity was quenched by acidifying with 2% (v/v) trifluoroacetic acid (TFA) to reach a final concentration of 0.2%. The digested peptides were desalted according to the in-StageTip (iST) method 24, using a single SDB-RPS disk, then dried by vacuum centrifugation, and resuspended in 2% ACN, 0.1% formic acid (FA) in water for liquid chromatography-mass spectrometry (LC-MS) analysis.

#### LC-MS/MS analysis

Peptide samples were analyzed using an UltiMate 3000 RSLCnano system coupled to a Q Exactive Plus mass spectrometer (Thermo Scientific). Peptides were separated on an EASY-Spray PepMap RSLC C18 column (50 cm × 75 μm, 2 μm particle size, Thermo Scientific) at a flow rate of 250 nL/min. A non-linear gradient of 2% to 45% solution B (80% ACN, 20% H₂O, 5% dimethyl sulfoxide (DMSO), 0.1% FA) over 50 minutes was used for elution, with solution A consisting of 0.1% FA in water. The mass spectrometer operated in positive polarity and data-independent acquisition (DIA) mode. Full MS scans were acquired in the Orbitrap analyzer over an m/z range of 375-1500 at a resolution of 70,000, with an automatic gain control (AGC) target set to 3×10^6^. For MS/MS fragmentation, precursors were isolated with a 34 m/z window and fragmented by higher-energy collisional dissociation (HCD) with a collision energy of 27%. MS2 scans were acquired at a resolution of 35,000, using an AGC target of 3×10^6^, with 19 loop counts for comprehensive precursor coverage.

The mass spectrometry proteomics data have been deposited to the ProteomeXchange Consortium via the PRIDE 25 partner repository with the dataset identifier PXD057842.

We have submitted all relevant data of our experiments to the EV-TRACK knowledgebase (EV-TRACK ID: EV250085) 26.

### Statistical analysis

Two-way ANOVA has been used for particle size distribution and RBCEP internalization by responder cells. Ordinary One-Way ANOVA has been applied for particle and protein concentration analysis and for ROS assay. RBCEP dimensions and surface functionalization by click chemistry have been analyzed by the Kruskal-Wallis test and multiple comparisons.

For proteomic analysis, raw files were processed using Spectronaut v18 (Biognosys AG) in directDIA mode under default settings. The library was generated from the Uniprot Human database (downloaded June 2024). Enzymes/Cleavage Rules were specified as Trypsin/P, LysC. Carbamidomethylation was selected as a fixed modification, while methionine oxidation and N-terminal acetylation were selected as variable modifications. The false discovery rate (FDR) of peptide spectrum match (PSM) and peptide/protein groups was set to 0.01. For quantification, Precursor Filtering was set to Identified (Qvalue) and MS2 was chosen as quantity MS-level. The Spectronaut Protein Quant Pivot Report was imported into Perseus software v1.6.5.0 27 for pre-processing. To ensure data quality, protein groups were filtered requiring at least five valid intensity values out of six in at least one experimental group. The remaining missing values were imputed column-wise using random values drawn from a normal distribution with a downshift of 1.8 and a width of 0.3, simulating low-abundance values close to the noise level. Downstream statistical analyses and data visualizations were performed using R Statistical Software v4.4.1. To identify differentially expressed proteins (DEPs) among the three experimental groups, a one-way ANOVA was conducted, followed by Benjamini-Hochberg (BH) correction to adjust p-values for multiple comparisons. Proteins with a BH-adjusted p-value < 0.05 were considered statistically significant DEPs. Post hoc analysis using Tukey's Honestly Significant Difference (HSD) test was then performed to identify specific pairwise differences between groups. The up-regulated proteins from each EV isolation method were analyzed for enrichment of Jensen COMPARTMENTS and Gene Ontology (GO) Molecular Function terms using the Enrichr 28 and ShinyGO v0.80 29 tools, respectively. Figures were generated using a combination of R packages, including ggplot2 for general plotting 30, ComplexHeatmap for heatmap visualizations 31, VennDiagram for Venn diagrams 32, and eulerr for Euler diagrams 33.

## Results

### NanoEs represent the largest RBCEP formulation and are produced in greater quantities compared to EryEs and VI-EryEs

**Figure 1:**
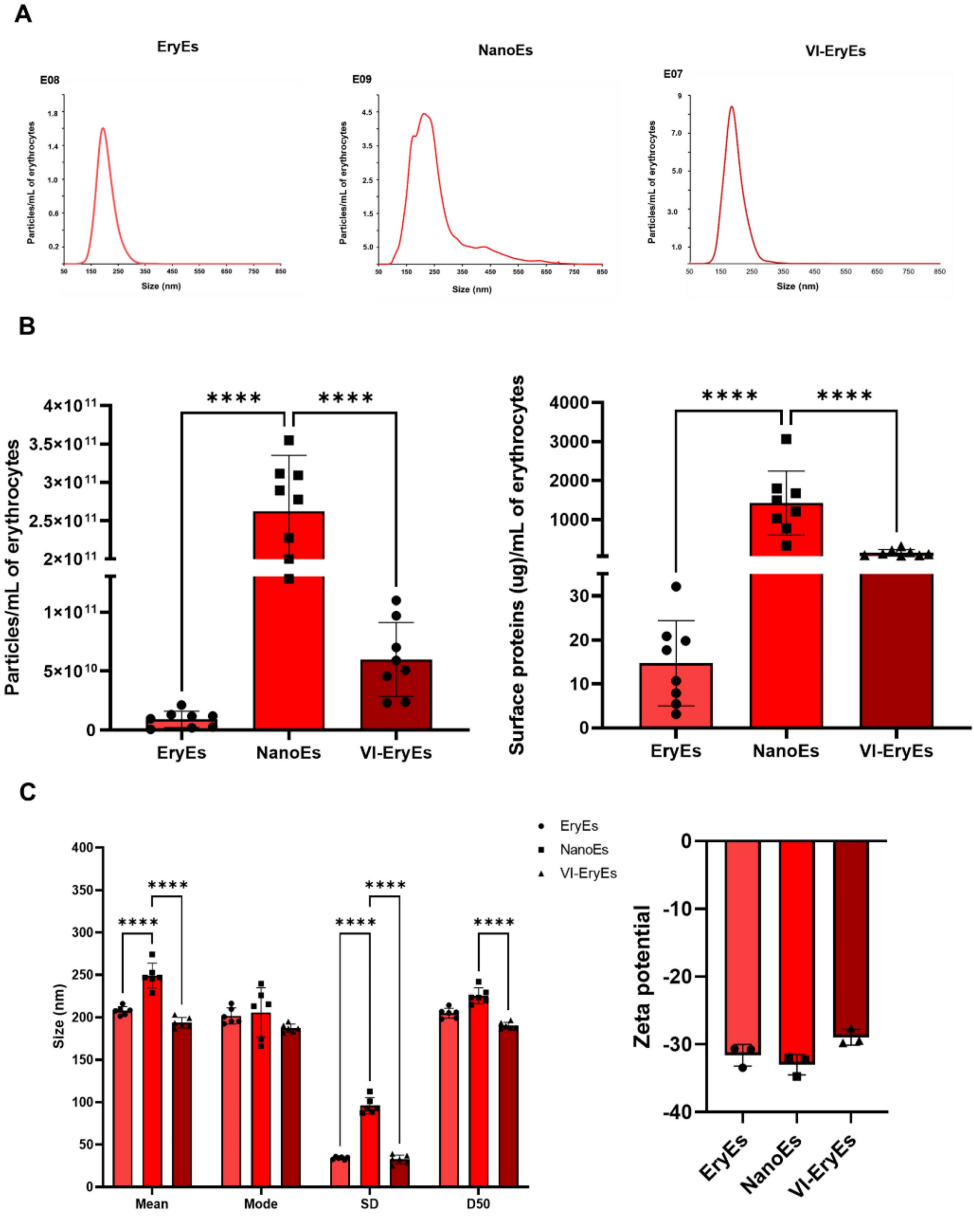
** RBCEP size distribution, concentration and zeta potential**. (A) Representative NTA measurements of the mean size distribution (*N* = 4) of each RBCEP subpopulation; (B) Left panel: histogram showing the number of particles isolated from 1mL of RBCs for each subpopulation (NTA) (N=8). Data are represented as mean ± SD. **** p<0.0001, (Ordinary One-Way ANOVA); right panel: histogram showing the number of proteins isolated from 1mL of RBCs for each subpopulation (BCA) (N=8). Data are represented as mean ± SD. **** p<0.0001, (Ordinary One-Way ANOVA).; (C) Left panel: mean values of the NTA dimensional parameters “Mean”, “Mode”, “SD”, “D50” (N= 6). Data are represented as mean ± SD. **** p<0.0001 (Two-way ANOVA); right panel: histogram showing the zeta potential of the three RBCEP formulations (N=3). Data are represented as mean ± SD.

Unlike NanoEs, both EryEs and VI-EryEs exhibited a monodisperse and comparable size distribution, as indicated by the NTA profiles (Fig. 1A). NTA analysis considered parameters such as median, mean, and span values to evaluate particle size and polydispersity. According to the manufacturer (Malvern Panalytical), a span value below 0.5 indicates a monodisperse suspension, while values above 1.0 denote polydispersity. EryEs and VI-EryEs had similar span values (0.39 ± 0.03 and 0.38 ± 0.04, respectively), confirming their monodispersity. In contrast, NanoEs showed a higher span value (1.00 ± 0.12), categorizing them as polydisperse. Particle concentration was assessed using two metrics: particles/mL of RBCs (Fig. 1B, left) and micrograms of surface proteins/mL of RBCs (Fig. 1B, right), both showing consistent trends. NanoEs were the most abundant subpopulation, yielding approximately 4 times more particles (2.62E+11 ± 7.29E+10 particles/mL) and 10 times more protein mass (1422 ± 820.5 µg/mL) than VI-EryEs (5.98E+10 ± 3.14E+10 particles/mL; 146.4 ± 83.32 µg/mL). Compared to EryEs (9.05E+09 ± 6.96E+09 particles/mL; 14.71 ± 9.7 µg/mL), NanoEs represented an even greater increase, about 29 times more particles and 97 times more protein mass (Fig. 1B). NanoEs also exhibited the largest particle size, with significantly higher mean (EryEs: 207 ± 5.1 nm; NanoEs: 249.2 ± 14.6 nm; VI-EryEs: 193.7 ± 6.2 nm) and median (D50) values (EryEs: 204.9 ± 5.9 nm; NanoEs: 225.4 ± 9.4 nm; VI-EryEs: 190.2 ± 3.9 nm) (Fig. 1C, left panel). Additionally, as evaluated with a Malvern Zetasizer, the zeta-potential of the three RBCEP formulations was found to be about -30 mV, that represents the typical range for extracellular vesicles (Fig. 1C, right panel).

**Figure 2:**
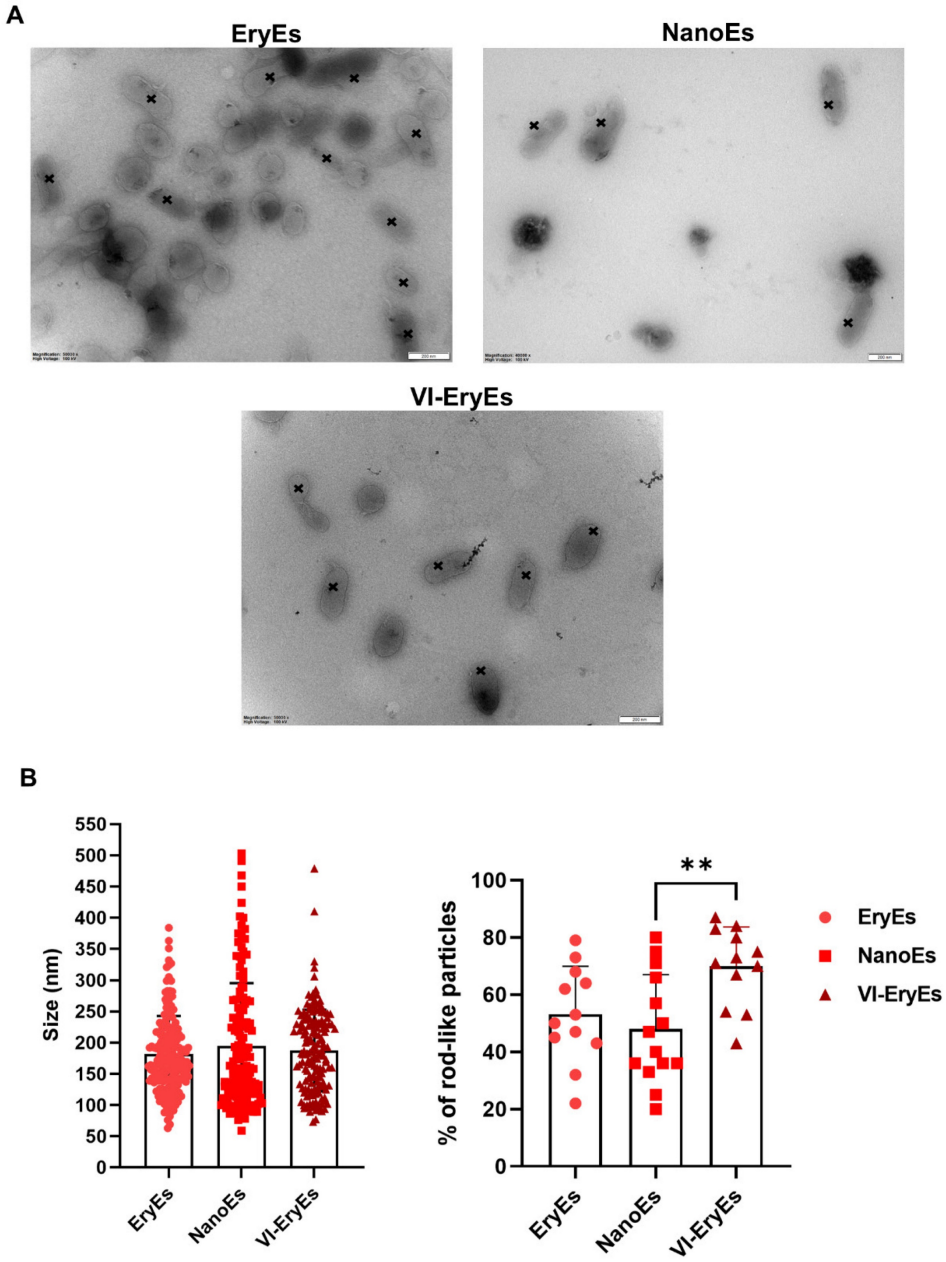
** RBCEP morphology and dimension**. (A) Representative TEM images of RBCEPs formulations; black crosses indicate the tubular shape in each subpopulation. (B) Histograms showing the mean size (left panel) and the percentage of rod-like particles in each subpopulation (right panel) . Data are represented as mean ± SD. **p<0.01 (Kruskal-Wallis test, Multiple comparisons).

We observed the coexistence of rod-like and traditional rounded shapes, with rod-like vesicles appearing in over 45% of all three analyzed populations (Fig. 2A and 2B, right panel). Notably, the proportion of rod-shaped VI-EryEs (70±12.7%) was significantly higher than that of NanoEs (48±19%), while no significant difference was detected between VI-EryEs and EryEs (53.17±18.8%) (Fig. 2B, right panel). TEM analysis confirmed that NanoEs were the largest population (194.6±100.7 nm), compared to EryEs (181.4±61.1 nm) and VI-EryEs (187.4±65.2 nm) (Figs. 2A and 2B, left panel).

Overall, these findings indicate that NanoEs exhibit the largest size, highest polydispersity, and greatest concentration, whereas EryEs and VI-EryEs are smaller, more uniform in size, and present at lower concentrations.

### All RBCEP formulations express Glycophorin a (CD235a) and the EV markers Alix, Flotillin-1 and Tsg101

**Figure 3:**
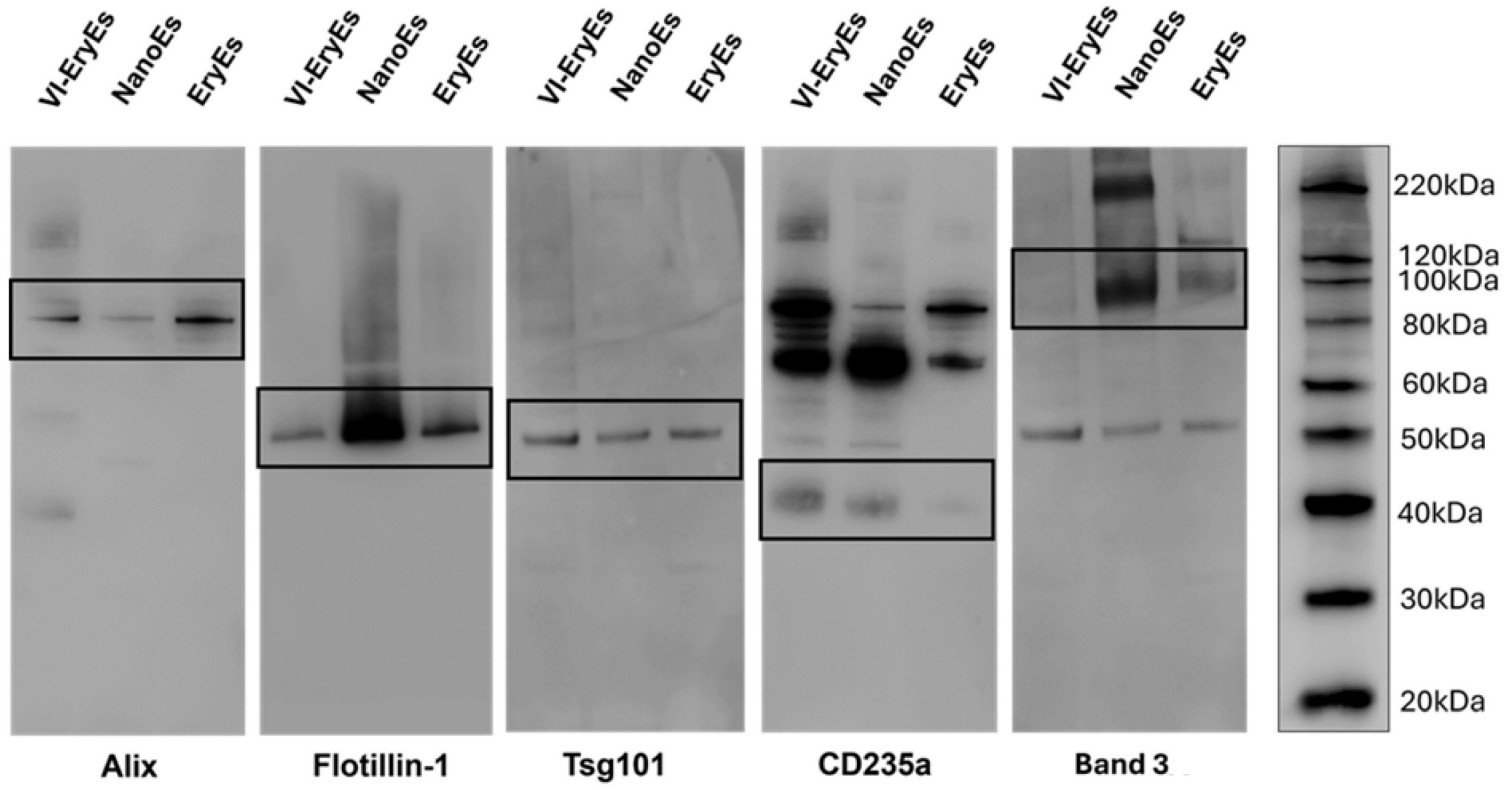
** RBCEP characterization**. Representative western blot indicating the expression of the EV markers Alix, Flotillin-1, Tsg101, and the presence of the RBC markers CD235a and Band 3 in each subpopulation.

The phenotypic profile, encompassing the expression of tetraspanins (CD9, CD63, CD81) and other EV-markers (Alix, Flotillin-1, and Tsg101), along with the two most common erythrocyte markers, CD235a (Glycophorin a) and Band 3, was analyzed by non-conventional flow cytometry (FCM) and western blot (WB) (Fig. 3, Supplementary Fig. 1 and Supplementary Fig. 2C). As expected, none of the three RBCEP formulations falling within the dimensional gate obtained after sample staining with CFSE (Supplementary Fig. 1A-C) expressed significant levels of the considered tetraspanins (Supplementary Fig. 1D-F). Flow cytometry revealed that the RBC marker CD235a was highly expressed across all subpopulations, with over 85% positivity (EryEs: 86.4 ± 11.4%; NanoEs: 95.7 ± 4.5%; VI-EryEs: 96.1 ± 3.7%), a finding also supported by western blot (Fig. 3). The vesicle markers Alix, Flotillin-1, and Tsg101 were expressed by all three groups (Fig. 3). Interestingly, Band 3 was consistently absent in VI-EryEs across all independent experimental replicates, in stark contrast to its clear expression in both EryEs and NanoEs (Fig. 3 and Supplementary Fig. 2C). These findings demonstrate that Alix, Flotillin-1, and Tsg101 serve as reliable markers for characterizing all three RBCEP formulations, while CD235a acts as the common erythrocyte marker shared by all groups.

### The three RBCEP formulations are defined by distinct proteomic signatures

**Figure 4:**
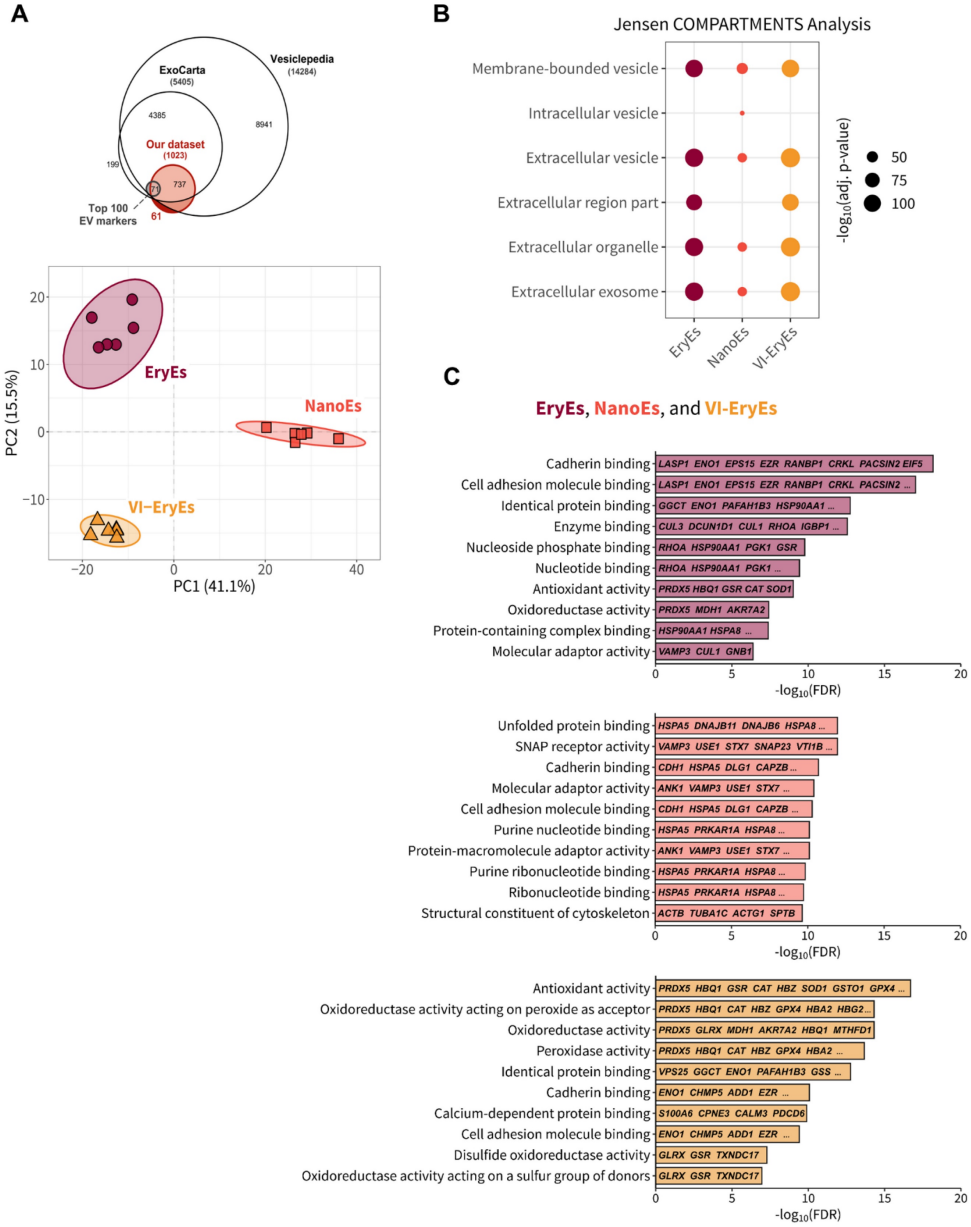
** Proteomic analysis of RBCEP formulations**. (A) Upper panel: Euler diagram illustrating the overlap of proteins identified in our analysis (n=1023) with established EV databases, such as ExoCarta (n=5405) and Vesiclepedia (n=14284), as well as the top 100 markers identified in EV studies from human tissues. Lower panel: Principal Component Analysis (PCA) plot showing a distinct clustering of EV proteomes based on isolation protocol. Each data point represents an individual replicate (n = 6 per group). 95% confidence intervals are indicated by shaded ellipses. (B) Results of the Jensen COMPARTMENTS enrichment analysis for upregulated proteins in each EV subpopulation, conducted using the Enrichr tool. The top five enriched subcellular compartments for each group are shown. Dot size reflects the significance of enrichment (-log₁₀ (adjusted p-value)), with larger dots indicating higher enrichment levels. (C) Gene Ontology (GO) Molecular Function enrichment analysis results for each EV subpopulation. Significantly upregulated proteins from each isolation protocol were analyzed using ShinyGO v0.80, and the top 10 enriched terms are displayed for each group. The x-axis shows -log₁₀(FDR) values, indicating enrichment significance, while the y-axis lists the GO terms. Gene names associated with each function are displayed within the bars, with each subpopulation (NanoEs, VI-EryEs, and EryEs) represented by distinct color-coded bars.

We performed an untargeted mass spectrometry (MS)-based proteomic analysis to characterize the protein composition of RBCEP formulations. A total of 1216 proteins were identified across all samples (n=6 for each subpopulation), with 1023 retained for analysis after excluding those with fewer than five valid intensity values per replicate. Of these, 71 matched the top 100 most frequently identified EV markers from the ExoCarta and Vesiclepedia databases, and 61 were novel (Fig. 4A, upper). Principal component analysis (PCA) revealed distinct proteome clustering for EryEs, NanoEs, and VI-EryEs (Fig. 4A, lower). Differential expression analysis identified 738 proteins with a Benjamini-Hochberg (BH)-adjusted p-value < 0.05. Post hoc pairwise comparisons using Tukey's test revealed distinct protein abundance differences between the subpopulations. An unsupervised hierarchical clustering analysis of the top 50 most significant differentially expressed proteins (DEP) confirmed the presence of three distinct proteomic profiles corresponding to each RBCEP formulation (Supplementary Fig. 3A). EryEs and VI-EryEs were more similar to each other than to NanoEs. A Venn diagram of upregulated proteins showed that 58% of the upregulated proteins in EryEs and VI-EryEs were shared, while 58% of those in NanoEs were unique to this subpopulation (Supplementary Fig. 3B). In particular, 356 proteins were found to be upregulated in NanoEs of which 79 were in common with VI-EryEs and 69 with EryEs; 89 were upregulated in EryEs of which 216 were in common with VI-EryEs and 77 proteins were upregulated in VI-EryEs (Supplementary Fig. 3B). These numbers demonstrated that a similar amount of protein is upregulated in EryEs and VI-EryEs (374 and 372 respectively) and 58% (the major part) of those are shared proteins between the two groups. Conversely, 58% of the upregulated proteins in NanoEs are unique in this subpopulation while only 19% and 22% are also upregulated in EryEs and VI-EryEs respectively (Supplementary Fig. 3B). Enrichment analyses of upregulated proteins revealed that all three subpopulations were enriched for membrane-bound vesicle, extracellular vesicle, organelle, and exosome-associated proteins, consistent with their vesicular origin (Fig. 4B). For subcellular localization, this analysis was performed using the "Jensen COMPARTMENTS" database via the Enrichr tool 28. For each subpopulation, we selected the top five most significantly enriched terms. EryEs and VI-EryEs had similar enrichment profiles, whereas NanoEs showed lower enrichment for these compartments. NanoEs did not express proteins associated with the extracellular region or intracellular vesicles, unlike EryEs and VI-EryEs (Fig. 4B). Gene Ontology (GO) Molecular Function enrichment analysis was performed using ShinyGO v0.80 29. The top 10 most significant terms were selected based on false discovery rate (FDR cutoff < 0.05) (Fig. 4C). NanoEs were enriched for binding (60%), molecular adaptor (30%), and structural activity (10%). VI-EryEs showed a predominance of catalytic (40%) and binding activities (40%), with notable antioxidant activity (20%). EryEs were mainly enriched for binding (70%), with minor antioxidant, catalytic, and molecular adaptor activities (Fig. 4C).

VI-EryEs uniquely exhibited catalytic and antioxidant activities, absent or minimal in NanoEs and EryEs. These results highlight distinct molecular functions across the RBCEP formulations.

### Enhanced membrane functionalization of NanoEs by click chemistry and RBCEP formulation safety

**Figure 5:**
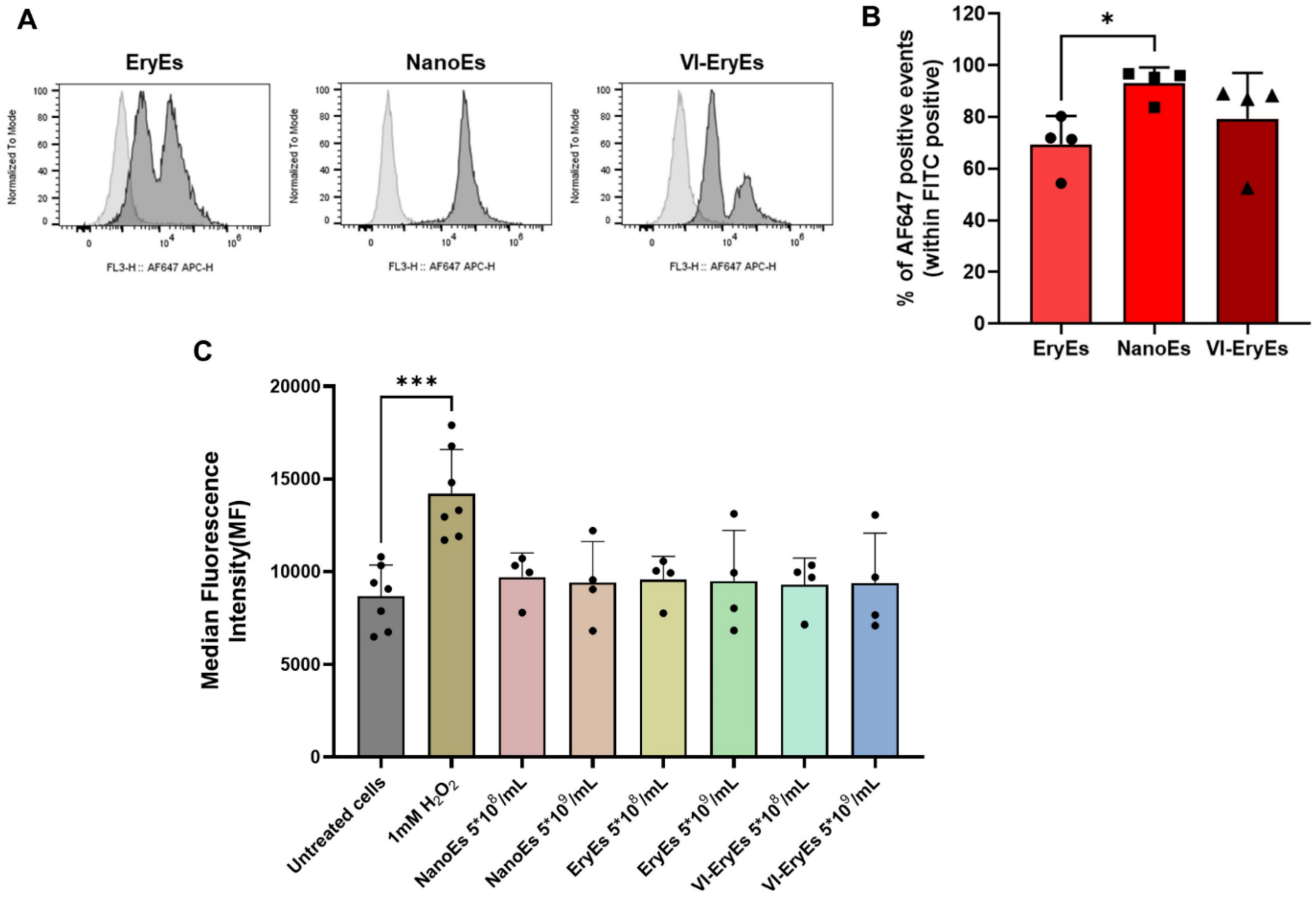
** RBCEP surface functionalization by click chemistry and total reactive oxygen species (ROS) production.** (A) Representative FCM of AF647-Click particles. The area under the gray line indicates the negative control (RBCEPs + AZ-647); the area under the black line represents the AF647-Click particles (RBCEPs + DBCO-NHS + AZ-647). (B) Percentage of APC-positive particles (Click-particles) in each group (N=4). Data are represented as mean ± SD. *p<0.05 (Kruskal-Wallis test, Multiple comparisons). (C) Median Fluorescent Intensity (MFI) associated with the production of ROS by MRC-5 cells.

RBCEPs have primarily been applied in drug delivery, with their membranes successfully functionalized for targeted therapeutic purposes using various strategies, including click chemistry 14,15. In this study, a validated copper-free click chemistry approach was used to functionalize RBCEP membranes with a fluorescent azide (AF647-azide), as previously described 20. Functionalization efficiency was assessed via FCM, revealing successful labeling in all three subpopulations, with over 70% AF647-positive particles in each group (EryEs: 69.5 ± 10.9%; NanoEs: 92.0 ± 6.2%; VI-EryEs: 79.2 ± 17.8%) (Fig. 5A). NanoEs demonstrated the highest suitability for membrane engineering, exhibiting a significantly greater proportion of AF647-positive particles compared to EryEs and VI-EryEs. To further evaluate the biocompatibility and inert nature of the different RBCEP formulations, we assessed the intracellular production of reactive oxygen species (ROS) by FCM analysis. MRC-5 cells (a fibroblast cell line) were incubated with EryEs, NanoEs, and VI-EryEs at two different concentrations (5*10^8^/mL and 5*10^9^/mL) (Fig. 5B and Supplementary Fig. 4). Hydrogen peroxide (1 mM) was used as positive control, and both conditions were compared to untreated cells (Fig. 5B and Supplementary Fig. 4). ROS production in recipient cells was calculated by evaluating the Median fluorescence intensity (MFI) in the FITC channel (Fig. 5B). A significant increase of MFI was observed upon H_2_O_2_ stimulation; on the other hand, either NanoEs, EryEs or VI-EryEs treatment was comparable with untreated cells (Fig. 5B). This additional* vitro* assay confirmed that none of the RBCEP types induced significant ROS production compared to untreated controls, indicating that the particle formulations are well tolerated and do not trigger oxidative stress. These findings, which are shared across all three RBCEP formulations, together with the enhanced surface functionalization ability that particularly characterizes NanoEs, provide strong support for their potential use as safe and effective drug delivery vehicles.

### Comparable internalization of different RBCEP formulations by responder cells

**Figure 6:**
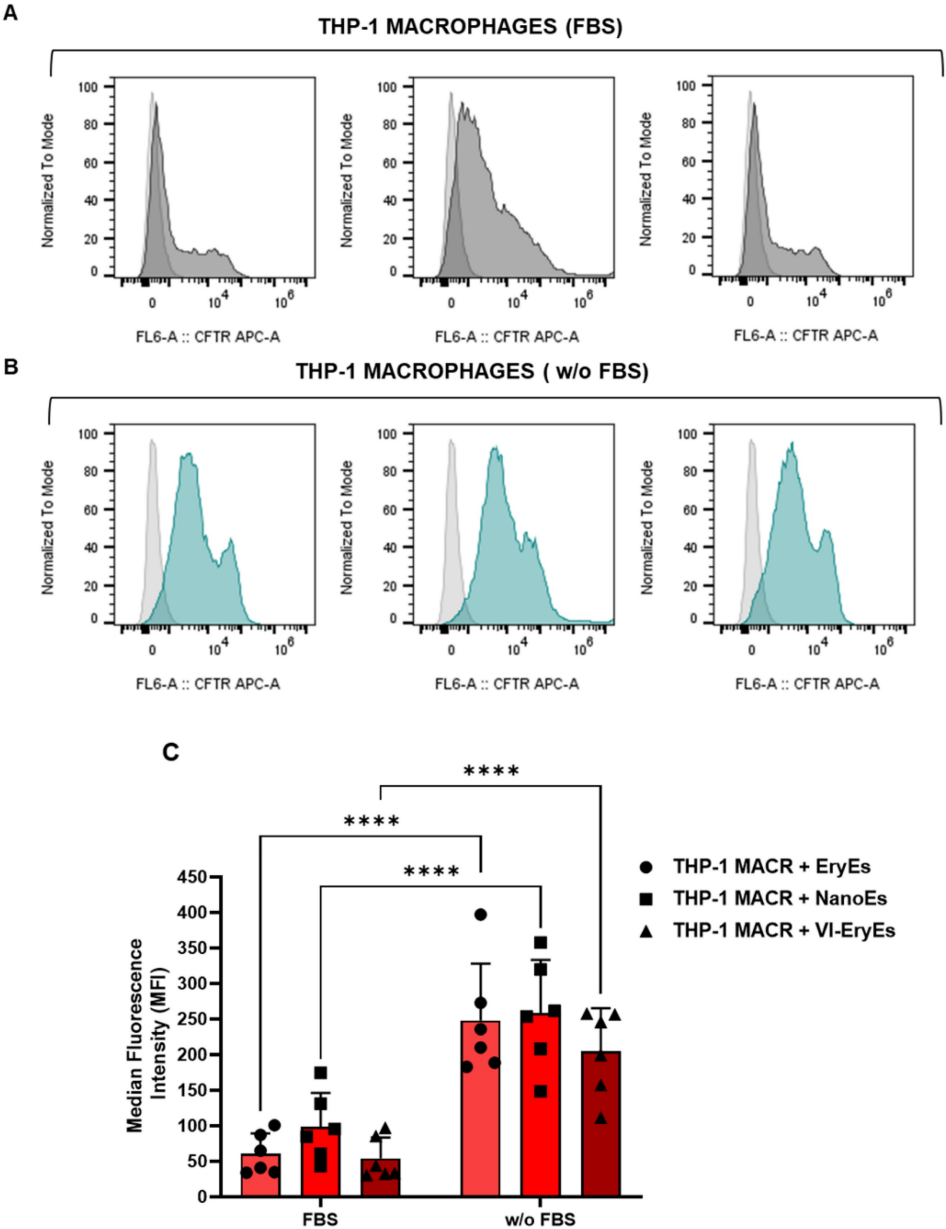
** RBCEP internalization by THP-1 macrophages**. (A) Representative FCM of THP-macrophages. The area under the gray line indicates untreated cells while the area under the black lines represents THP-1 macrophages treated with EryEs (left panel), NanoEs (middle panel), or VI-EryEs (right panel) in the presence of FBS-completed medium. (B) Representative FCM of THP-macrophages. The area under the gray line indicates the untreated cells while the area under the turquoise lines represents THP-1 macrophages treated with EryEs (left panel), NanoEs (middle panel), or VI-EryEs (right panel) in the presence of FBS-depleted medium. (C) MFI associated with «Cell Trace Far Red (CTFR) Proliferation Kit» stained particles internalized by cells with or w/o FBS (N=6). Data are represented as mean ± SD. ****p<0.0001 (Two-way ANOVA).

The uptake of CFTR-stained EryEs, NanoEs, and VI-EryEs by responder cells was evaluated in a macrophage cell line (THP-1) and a fibroblast cell line (MRC-5) to assess internalization differences (Fig. 6 and Supplementary Fig. 2A, B). Proper particle labeling with APC-conjugated CFTR dye was confirmed via FCM (Supplementary Fig. 2A, B, left panel). Cells were treated with defined RBCEP amounts for 3 hours in media with or without fetal bovine serum (FBS), which mimics physiological conditions. Untreated cells served as baseline control.

Internalization rates were calculated by comparing the normalized Median Fluorescence Intensity (MFI) across experimental groups (Fig. 6C and Supplementary Fig. 2B, right panel). Consistent with prior reports 15, FBS reduced particle internalization significantly (e.g., EryEs with FBS vswithout: 60.5±28.6 vs. 247.9±80.3 for THP-1; 2574.4±1409.4 vs. 5021.0±2252.3 for MRC-5). Similar trends were observed for NanoEs and VI-EryEs. However, no significant MFI differences emerged among EryEs, NanoEs, or VI-EryEs under identical conditions (with or without FBS), indicating that internalization depends on FBS presence rather than inherent subpopulation properties, a pattern consistent across both cell lines.

## Discussion

The innate role of intercellular communicators and the natural ability to encapsulate and protect specific cargoes have made extracellular vesicles an attractive tool for targeted drug delivery. Precision and safety are the criteria that guided the decision to demonstrate the potential of these bio-derived products 34 in being engineered and used in therapeutic applications, particularly in cancer therapy. In this sense, the evidence of their efficacy has been widely demonstrated over the last few years. EVs from diverse sources have shown success in cancer immunotherapy and drug delivery across *in vitro*, *in vivo*, and clinical settings 34. Since EVs inherit the unique properties of their source cells, selecting the appropriate source is crucial for specific applications. Erythrocytes offer significant advantages as extracellular particle (EP) producers, including their abundance, ease of isolation, lack of a nucleus, and primary role as efficient transporters in the bloodstream. These attributes translate into exceptional potential for erythrocyte-derived EPs as drug delivery vehicles, a utility well-documented in healthcare and clinical contexts 3,11,13,15. However, their intrinsic therapeutic properties have just started to be explored 35,36. Significant efforts have been made recently to optimize protocols for their isolation in order to harness their potential. To date, isolation methods for these vesicles can be broadly classified into three categories: vesicles naturally released into storage bags during erythrocyte preservation, those induced by chemical stimulation, and those generated through physical stimulation of erythrocytes.

Considering the variety of methods available for isolating RBCEPs, their characteristics may vary, potentially affecting their efficiency as drug delivery vehicles. Consequently, their functionality could be influenced by the isolation method used. To the best of our knowledge, no study has yet evaluated the properties of RBCEPs produced by the three before-mentioned strategies. In this study, we assessed the quantitative and qualitative differences among three formulations of RBCEPs, corresponding to the three isolation methods: Nanoerythrosomes (NanoEs), obtained by sonication of erythrocytes; Eryerythrosomes (EryEs), naturally released during erythrocyte preservation; and Vesiculation-induced Eryerythrosomes (VI-EryEs), generated through calcium ionophore stimulation. We also evaluated their potential for surface engineering using a previously described copper-free click chemistry approach 20, based on our prior demonstration that the amount of EPs is critical for successful membrane functionalization. Our initial comparison focused on correlating EP concentration with particle size and distribution. NanoEs were distinct from the other subpopulations, showing the largest size, higher concentration, and a polydisperse distribution, while VI-EryEs and EryEs exhibited monodisperse distributions and were more similar in terms of size and yield. Morphological analysis revealed the presence of rod-like or "tubular-shaped" particles alongside the traditional rounded shape in all subpopulations, with VI-EryEs showing the highest percentage of rod-like particles. This tubular morphology has been previously described in EryEs 37 and is similar to the elliptocyte shape observed in erythrocytes under conditions like hereditary elliptocytosis, thalassemia, and storage lesions in RBCs 38,39, 40-42. Therefore, the elliptical shape in RBCEP samples may reflect the formation of elliptocytes due to storage-related changes in RBC deformability. The three RBCEP formulations were then characterized for EV and erythrocyte marker expression by non-conventional flow cytometry and western blot. As previously reported, RBCEPs do not display EV surface markers such as CD9, CD63, and CD81 17, and we have confirmed that these tetraspanins are absent in all three RBCEP formulations.

However, other EV markers, including Tsg101, Flotillin-1, and Alix, were present. Glycophorin A (CD235a) was universally expressed across all subpopulations, while Band 3 was absent only in VI-EryEs. This is in contrast with previous observations showing the expression of Band 3 in RBC-derived EVs produced upon calcium ionophore stimulation 43,44. Sample handling, storage time (both cited studies employ fresh RBCs compared to the 42-day old erythrocytes used in this work) and differences in protocols for isolating RBCEPs may be the possible reason behind this discrepancy. In our case, we suggest that the long-term storage affecting RBCEP characteristics could increase their susceptibility to calpains which are activated by calcium treatment 45 so leading to Band 3 degradation. This event was demonstrated to be critical for erythrocyte membrane integrity and cell shape maintenance 45-49. Therefore, the loss of Band 3 in VI-EryEs may explain their higher proportion of elliptical-shaped particles which in turn have not been observed in Band 3 expressing calcium ionophore-induced RBCEVs.

An in-depth proteomic analysis of the three RBCEP formulations was conducted, comparing the identified proteins with vesicular protein databases ("ExoCarta" and "Vesiclepedia"), revealing significant overlap as well as novel proteins not previously reported. Principal component analysis (PCA) showed distinct proteomes for each subpopulation, confirming unique protein expression profiles. Differential expression analysis identified 738 differentially expressed proteins (DEPs), with EryEs and VI-EryEs sharing more upregulated proteins than NanoEs. To further investigate their origin and function, we performed two additional analyses: (i) "Jensen COMPARTMENTS" for subcellular localization and (ii) "Gene Ontology (GO) Molecular Function" enrichment. The first analysis revealed high enrichment in all three subpopulations for proteins typically found in membrane-bounded vesicles, extracellular vesicles, organelles, and exosomes, confirming their origin from these compartments. Notably, NanoEs exhibited distinct enrichment patterns compared to EryEs and VI-EryEs. The GO molecular function analysis revealed that NanoEs and EryEs were more similar to each other than to VI-EryEs, with "binding activity" being the most common function across all groups. VI-EryEs, however, showed a distinct enrichment for "antioxidant activity," while "molecular adaptor activity" was present in NanoEs and EryEs but absent in VI-EryEs. These findings suggest that the different RBCEP formulations may engage in distinct molecular pathways, offering diverse biological applications. Targeted drug delivery is the primary application for these particles, making their ability to undergo engineering a crucial property. We functionalized the EP surface with a fluorescent azide using a previously described copper-free click chemistry approach 20. NanoEs exhibited the highest functionalization efficiency, likely due to their larger size, which provides more available binding sites on the surface. This, along with their higher yield and ease of isolation, positions NanoEs as the most promising candidates for targeted delivery. RBCEP safety was also assessed by examining the intracellular production of reactive oxygen species (ROS) after treating MRC-5 fibroblasts. The amount of ROS production was comparable to untreated cells for all three RBCEP formulations thus demonstrating their tolerability. We also evaluated the uptake of all three subpopulations by MRC-5 fibroblasts and THP-1 macrophages, assessing internalization in the presence or absence of FBS. Internalization was not significantly influenced by the subpopulation type but was instead dependent on FBS presence, consistent with previous findings showing its negative impact on EP internalization 15. To further explore the biological activities and functions of these formulations, additional *in vitro* experiments using appropriate cell models based on proteomic analysis would be beneficial. Moreover, analyzing extracellular particles derived from RBC units stored for different durations would provide deeper insight into their intrinsic and biological properties, as well as how these characteristics may change depending on the storage time and the age of erythrocytes.

## Conclusions

Erythrocyte-derived particles are increasingly prominent in extracellular vesicle studies as leading candidates for pharmaceutical delivery, with their potential and efficacy widely demonstrated. Given the considerable efforts invested in developing standardized and high-yield isolation methods for these particles, it is also important to assess whether such methods may influence the vesicles' intrinsic properties. Our analysis of the differences among three distinct erythrocyte-derived EP formulations represents a pioneering effort in the in-depth study of these particles. As the first investigation of its kind, it opens new avenues for understanding not only their drug delivery potential but also their inherent biological effects.

## Supplementary Material

Supplementary figures.

## Abbreviations

RBCEPs: RBC-derived extracellular particles
EryEs: Eryerythrosomes
NanoEs: Nanoerythrosomes
VI-EryEs: Vesiculation Induced Eryerythrosomes
