# Supplementary material for: Impact of the production method on the properties of erythrocyte-derived extracellular particles: a quantitative and qualitative evaluation
Source: Nanotheranostics. 2025 Oct 24;9(3):299–314. doi: 10.7150/ntno.116627 (PMC12595265; doi:10.7150/ntno.116627)

Supplementary Figure 1. (A-C) Flow cytometry strategy used to characterize RBCEPs. Representative upper panels indicate EryEs (A), NanoEs (B), or VI-EryEs stained with CFDA-SE at 4°C as a "blank tube" to define the appropriate dimensional gate. The corresponding lower panels indicate the same subpopulations stained with CFDA-SE at 37°C. (D-F) Representative FCM showing the expression of CD9 (red lines), CD81 (blue lines), CD63 (green lines), and Glycophorin A (CD235) (violet line) for EryEs (D), NanoEs (E) and VI-EryEs (F). Areas under the gray lines indicate the expression of the corresponding isotype immunoglobulin. *Y*-axis scaling: channels have been scaled as a percentage of the maximum count (modal setting, FlowJo_v10.9.0).

Supplementary Figure 2. Representative dot plots indicating the strategy adopted to stain RBCEPs with CTFR. The dye in PBS represents the "blank tube" to define the appropriate dimensional gate (A, left panel). A-middle and right panels and B-left panel represent RBCEPs stained with the same dye. The histogram shows the Median Fluorescence Intensity (MFI) associated with CTFR-stained particles internalized by MRC-5 cells cultured in FBS presence or absence (B, right panel). Data are represented as mean ± SD, n=6. **p<0.01 (Twp-way ANOVA). (C) Western Blots indicating the expression of the EV markers Alix, Flotillin-1, Tsg101, and the presence of the RBC markers CD235a and Band 3 in each formulation (n=4).

Supplementary Figure 3. (A) Heatmap of the top 50 differentially expressed proteins (DEPs) across the three RBCEP subpopulations (NanoEs, VI-EryEs, and EryEs). Unsupervised hierarchical clustering was performed on the z-scored intensity values for each protein, with rows representing proteins and columns representing individual samples. Proteins that are significantly upregulated in specific subpopulations are annotated on the left side of the heatmap, with color-coded bars indicating upregulation in NanoEs (red), VI-EryEs (orange), or EryEs (purple). (B) Venn diagram illustrating the overlap of the 738 differentially expressed proteins (DEPs) among the three RBCEP subpopulations. Each circle represents the proteins upregulated in one subpopulation: NanoEs (n = 356), VI-EryEs (n = 372), and EryEs (n = 374). The overlapping areas indicate proteins shared between subpopulations, with the specific numbers of shared and unique DEPs displayed in each section.

Supplementary Figure 4. Total reactive oxygen species (ROS) assay. (A) Representative flow cytometry dot plots (FSC-A vs SSC-A) of MRC-5 cells untreated (Black), treated with 1 mM H_2O_2 (blue), with NanoEs (red), EryEs (light blue), or VI-EryEs (green) at two different concentrations (RBCEPs/mL). (B) Representative FCM histograms showing the same experimental conditions and the Blank condition that wasn't stained by ROS Assay Stain Solution.

Supplementary Figure 1

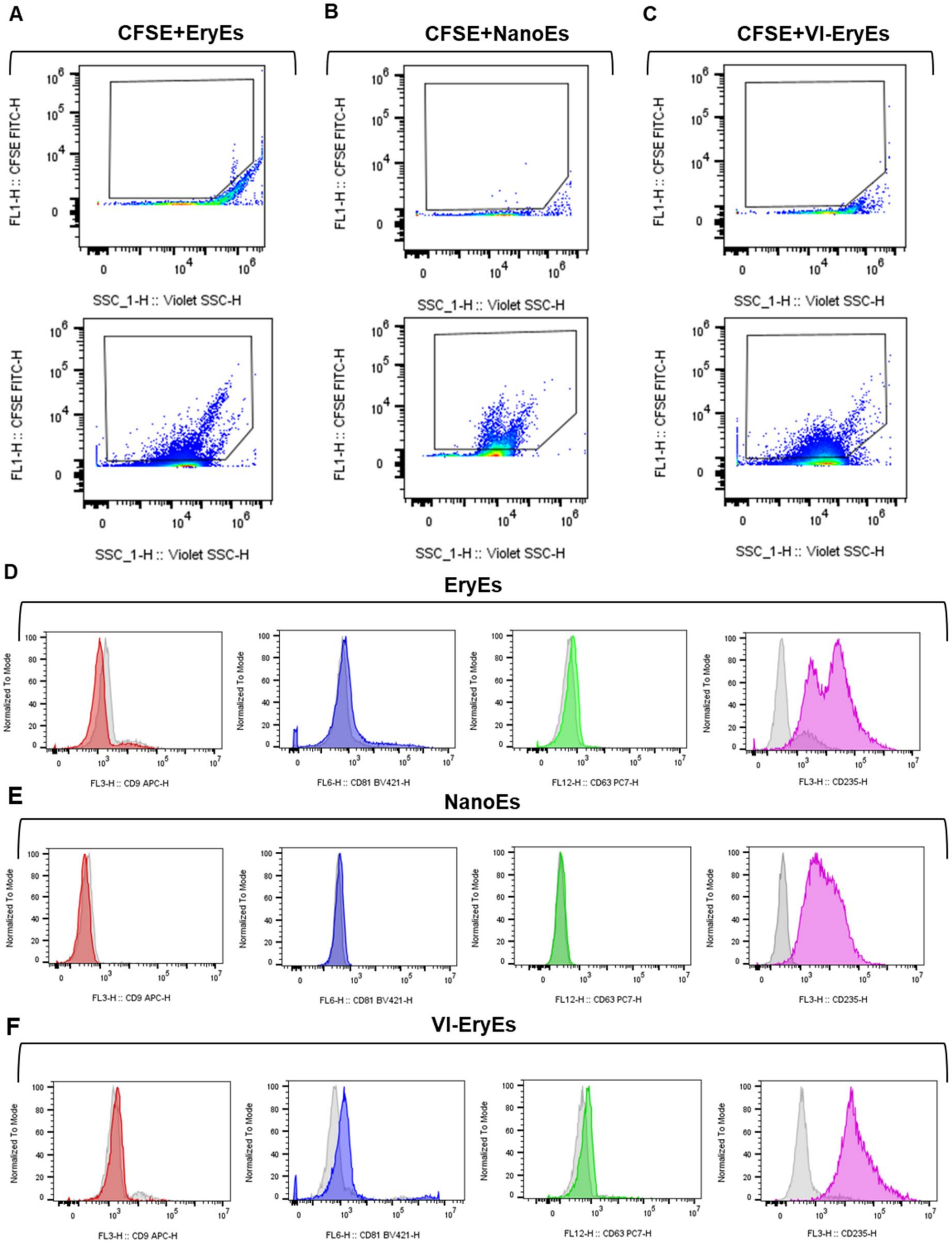

Supplementary Figure 2

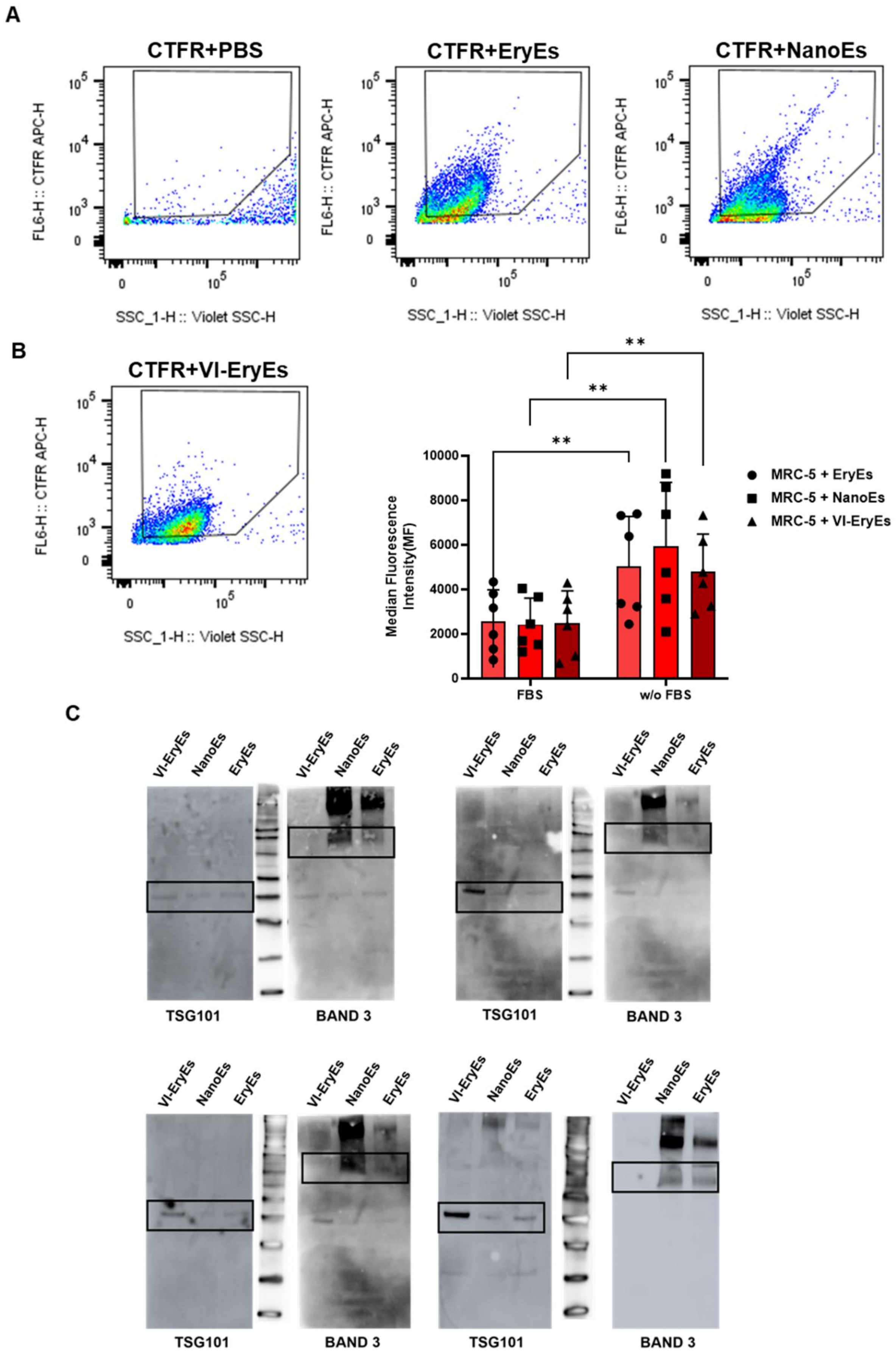

Supplementary Figure 3

A

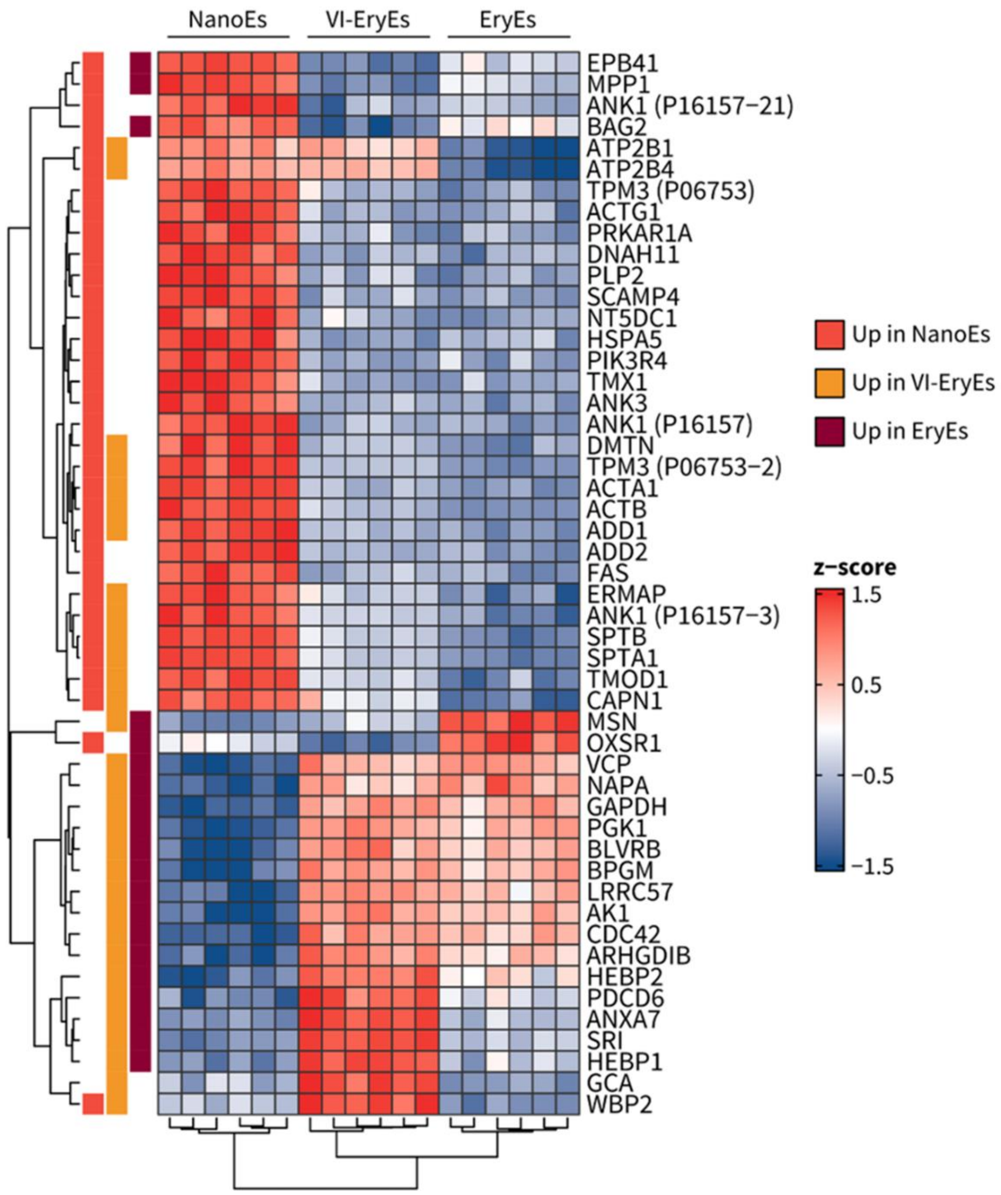

B

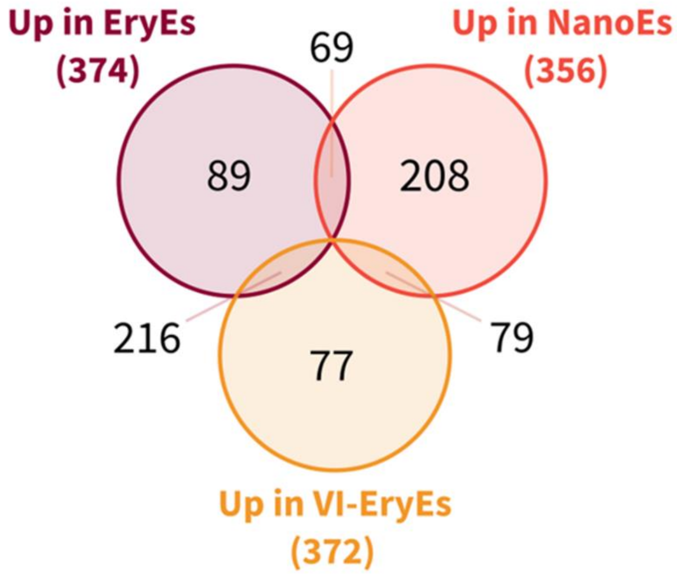

Supplementary Figure 4

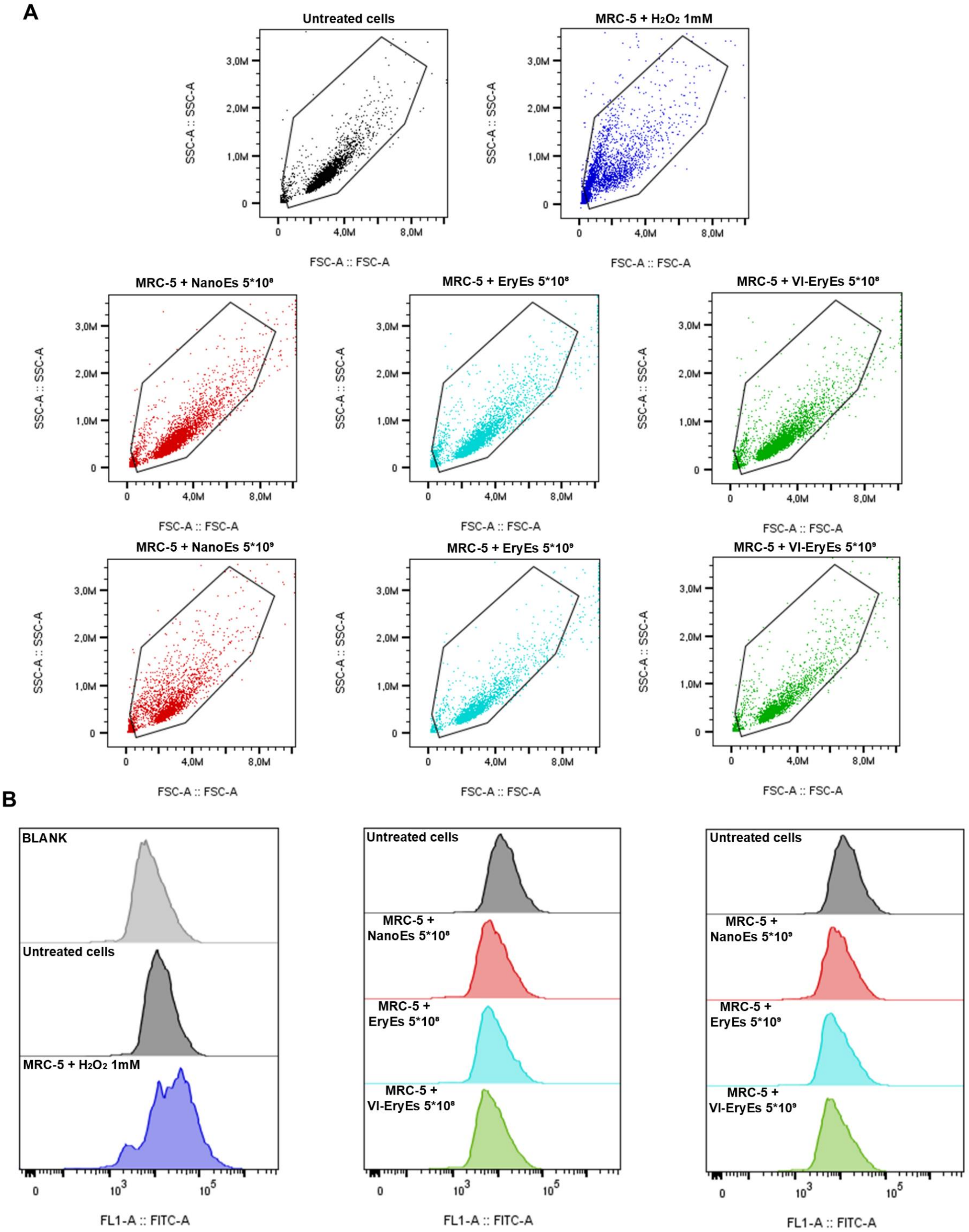

Supplement: Supplementary file 1 — Supplementary figures. [file ntnov09p0299s1.pdf]
